# On Snuff-Taking

**Published:** 1824-12

**Authors:** 


### Art. V.—*On Snuff-taking*. By Dr. Kinglake.

The practice of snuff-taking is, perhaps, the most baneful that popular custom and familiarity have sanctioned as innoxious and gratifying. It is scarcely conceivable to what an extent of misapprehension and fallacy, an authorised and an unsuspected habit may lead. It has rarely occurred to those who use snuff, even the most largely, that it is an agent possessing qualities that cannot fail to prove highly deleterious to the healthy tone and energy of the stomach.

Tobacco, of which snuff is the comminuted division or powder, is undeniably amongst the most powerful class of narcotic substances, and, were it to be taken into the stomach freely, it would exert an influence not less overwhelming and destructive than that which would arise from henbane, wolfsbane, deadly nightshade, hemlock, &c. In the whole tribe of narcotic vegetables, perhaps there is not one that would derange the healthy state of the stomach more deeply and seriously than tobacco.

Henbane, aconite, blue monkshood or wolfsbane, deadly nightshade, dogs-mercury, thorn-apple, common hemlock, bug agaric, pepper agaric, hemlock dropwort, water hemlock, laurel, &c. are severally resorted to as powerful medicines, designed to fulfil curative indications of extraordinary difficulty, but it never entered into the imagination of the most adventurous, to use either of these substances in the form and manner of snuff. When medicinally introduced into the stomach, it has been done in doses cautiously restricted, so as to prevent the possibility of any disastrous effect resulting from them. Were they familiarly taken like powdered tobacco or snuff, the most pernicious consequences would probably arise from such hazardous practice.

Snuff-takers do not advert to the route into which the noxious article is forced by the act of strong inhalation through the nostrils. It is neither supposed nor intended to pass beyond the anterior cavities of the nose; instead of which, it is carried through its posterior openings, commonly into the gullet; from thence, it finds its way into the stomach, and occasionally a portion will be apt to escape under the epiglottis into the trachea. In either case, immediate and distant mischief of a very afflicting nature is likely to ensue.

The stomach can no more decompose powdered tobacco, so as to render it comparatively harmless, than it can deadly nightshade, hemlock, or any other vegetable poison. It must therefore first disorder its healthy function, inducing dyspeptic ailment, morbid sensibility, and of course an endless train of distempered nervous feelings. The direct influence of tobacco on the stomach is in a high degree enervating, by which that organ is incapacitated for a healthy secretion of the gastric fluid, and for exerting the vital energy that is requisite for performing its digestive function. Loss of appetite, distressing sickness, gastric oppression, præcordial anxiety, acetous fermentation, flatulent distention, and deathly langour, are amongst the direct effects of admitting snuff into the stomach. The more distant injury which this hurtful agent is likely to occasion, may be perceived in the various sympathetic disturbance which a disordered stomach awakens throughout the whole system.

What vital function can preserve its healthy state amidst such overwhelming affection of gastric excitability?

The detriment arising to the stomach in the first instance, and secondarily to the system at large, is most insidious. The evil is incessantly working its mischievous course, without being at all suspected. In each succeeding portion of powdered tobacco that is incautiously snuffed through the nostrils, additional occasion is given for fresh and increasing injury, until at length dyspepsia and various nervous sensations accrue, which are familiarly attributed to any cause rather than to the real one. To impeach a favourite indulgence,—to charge with the cause of disease that which is held to be a harmless and gratifying excitement of the nasal cavities, would be regarded as wildly visionary, and wholly inadequate to such an effect. In this persuasion the use of the noxious agent is habitually and fearlessly pursued, until its morbific effects become sufficiently manifest to awaken serious apprehension.

Were either of the narcotic substances that have been enumerated, to be prepared for use as snuff, by the addition of such attenuating and stimulating ingredients as would fit it for pleasingly exciting the nostrils, the admission of the narcotic agent in this insidious manner into the stomach, would most likely very soon produce effects not dissimilar to those of tobacco. An article capable of operating on the living power of the stomach, like tobacco or any of the other active narcotics, cannot with safety be used as an inoffensive stimulant on a surface that has a direct and ready communication with the cavity of that organ, without incurring consequences of a very threatening nature. The magnitude of the mischief produced by this unknown or neglected cause is often irreparable. Prostrated, agitated, and variously distempered nervous power cannot be easily extricated and released from its almost paralised disabilities. Direct remedies for such ailments are not at hand, the poison is without an adequate antidote, and this consideration should seasonably warn the amateurs, the proficients, and the veterans in a practice alike uncleanly and unhealthy, to desist from it before it shall have incurably distempered and disorganised the structural as well as functional integrity of the stomach.

Rigid, absolute, and uncompromising abstinence from the pernicious custom of taking snuff, is the only preventive remedy of the numerous and often irremediable evils attending the indefensible usage. It probably under no circumstances could prove beneficial, whilst its injurious effects are various and frequently most embarrassing. It occasionally distempers the healthy action of the mucous membrane of the nostrils, inducing excoriations, polypous excrescences, and even ill-conditioned sores, that may assume the irrestrainable extension and character of cancerous virulence.

Many instances have fallen under my notice, and more occur to my reflection, of but little short of mortal injury having accrued from a profuse and an incautiously violent mode of forcing snuff through the nostrils into the gullet and stomach. Morbid changes in the structure of the posterior fauces and in different portions of the œsophagus, occasioning diseased contraction of that tube, have been justly referable to the distempering influence of tobacco. The worst cases of indigestion and of mesenteric atrophy have been reasonably supposed to have originated from excessive chewing and smoking as well as snuffing of tobacco, in which, a negative remedy has been found in the discontinuance of the practice.

There is much reason for believing that the ever-memorable Napoleon Bonaparte derived the cause of his protracted suffering and eventual death, from the large quantities of snuff which he lavishly but unconsciously carried into the stomach through the nostrils, by the habit of strong and unmeasured inspiration with which he used that destructive agent. The diseased appearances of the stomach on inspection after death, termed cancerous, were those of an highly inflamed, much thickened, and extensively ulcerated surface, such as were very likely to have been induced by the noxious influence of tobacco, almost incessantly supplied by the frequent, abundant, and forcible manner in which that illustrious personage was notoriously known to take that powdered article.

There can be no more valid reason assigned for persisting in the undeniably hurtful custom of taking snuff, than there could be for that of any other poison; and whoever will inconsiderately incur the imminent risk of occasioning irremediable and destructive mischief by so baleful a practice, will find no admissible excuse either in the prevalence of the custom, in its unobjected currency, or in the transient gratification and notional benefit attending its use.

If strongly exciting the mucous membrane of the nostrils can be supposed, from its proximity to the brain, to produce a beneficial effect on that organ, the purpose may be answered by substances not less pungent than tobacco, and without any of its deleterious qualities. The effluvia of ammonia in either a solid or liquid form, the aroma of pepper, ginger, or any other simple stimulant, mixed with either powdered chalk, liquorice, or cinnamon, in such proportion as would render the composition sufficiently powerful moderately to irritate, without excoriating the nasal membrane, would be an adequate substitute for what may be regarded as the harmless agency of tobacco, with a secure exemption from its pernicious influence. It is however not probable that the local excitement of the nostrils can ever prove salutary or advantageous, beyond the momentary gratification connected with the established habit of the practice; and as all unnecessary usages are rather nuisances than benefits, it would seem to be indispensably advisable to abstain from a custom that is unsightly in its appearance, preposterous in its observance, and in every conceivable view that can be taken of its effect, much more likely to become eventually injurious than useful.

As errhines, in sternutative intentions, various substances have been used for the purpose of exciting the minutely ramified expansion of the olfactory nerves on the mucous membrane of the nostrils. In cases of unyielding lethargy and comatose stupor, arising from cerebral oppression and other states of distempered sensibility, nasal stimulants may beneficially cooperate with suitable depletion in restoring nervous depression to a condition of natural freedom and energy. The close vicinity of the nostrils to the brain, with the direct nervous communication subsisting between those surfaces and that organ, explains the possibility of its being powerfully affected by remedies capable of provoking the concussive action of sneezing.

The most efficient of those medicinal substances are asarabacca (asarum europæum), herb mastick (teucrium marum), white hellebore (helleborus albus), yellow subsulphate of quicksilver (subsulphas hydrargyri flavus). Small quantities of either of these articles conveyed up the nostrils in instances of lethargic or apoplectic insensibility, in protracted syncope, and suspended animation, may be advantageously employed; and in attempting to fulfil such an indication of relief the design is purely medicinal, having no affinity to snuffing tobacco, and of course furnishing no warranty for that fashionable but offensive and reprehensible practice. The unnecessary use of errhines would occasion an unhealthy afflux of fluids to the nostrils, which would be at least a source of annoyance, if not of positive ailment. But the recited errhines have no narcotic or any other quality like tobacco, by which serious injury may be extended to the stomach, and more or less directly to the whole nervous system. In the use of snuff, it is less the stimulant impression on the nostrils than the transmission of the exciting substance to the gullet and stomach, that is denounced as mischievous, and reprobated as inadmissible.

*Taunton, August 15th,* 1824.

